# Low musculoskeletal health literacy is associated with inferior outcomes in total shoulder arthroplasty

**DOI:** 10.1016/j.jseint.2026.101677

**Published:** 2026-03-05

**Authors:** Jackson S. Perry, Bradley J. Lauck, Jake M. Reed, Alexander D. Jeffs, Stephen M. Himmelberg, Robert A. Creighton, Arvind S. Narayanan, Ganesh M. Kamath

**Affiliations:** a Department of Orthopaedics, The University of North Carolina School of Medicine, Chapel Hill, NC, USA; b The University of North Carolina, School of Medicine, Chapel Hill, NC, USA

**Keywords:** Musculoskeletal health literacy, Total shoulder arthroplasty, Survey outcomes, Patient satisfaction, Patient reported outcomes, Health disparities

## Abstract

**Background:**

The aim of this study was to investigate the impact of musculoskeletal (MSK) health literacy on clinical outcomes and patient satisfaction following total shoulder arthroplasty (TSA). We hypothesized that low MSK health literacy score would be associated with inferior clinical outcomes and lower patient satisfaction rates.

**Methods:**

A retrospective cohort study was conducted at a tertiary care center. Patients between 6 months and 9 years post-operative following shoulder arthroplasty, both anatomic total shoulder arthroplasty and reverse total shoulder arthroplasty were included. All patients were treated by one of 2 sports fellowship–trained surgeons. Patients were emailed a questionnaire that included basic demographics, American Shoulder and Elbow Surgeons (ASES) shoulder score, TSA satisfaction, and the Literacy in Musculoskeletal Problems survey. Patients were categorized as having low health literacy (LHL) if they answered fewer than 6 out of 9 questions correctly on the Literacy in Musculoskeletal Problems survey and as having normal health literacy (NHL) if they answered 6 or more questions correctly. Statistical analysis was performed to determine associations between LHL, patient characteristics, and outcomes of interest. This included multivariate logistic and linear regression models that assessed the relationship between MSK health literacy and ASES.

**Results:**

Of 734 patients emailed the survey, 310 responded, and 230 met inclusion criteria and were included in the final analysis. One hundred and seventy-seven patients (77%) had NHL and 53 individuals (23%) had LHL. The mean ASES score was 74.2 ± 19.8 in the NHL group and 62.9 ± 23.9 in the LHL group. Patients with LHL had significantly lower ASES scores than those with NHL (*P* = .0007). Patient satisfaction rate (90.3% vs. 82.7%, *P* = .2101) and willingness to undergo surgery again (89.8% vs. 84.9%, *P* = .334) were similar between the NHL and LHL groups, respectively. Multivariate analysis showed that NHL was independently predictive of a significantly higher ASES score (*P* = .009, odds ratio: 1.025).

**Conclusion:**

Approximately one-quarter of patients undergoing TSA at our institution had LHL. Patients with LHL were found to have significantly lower patient-reported outcome scores, although rates of patient satisfaction and willingness to undergo surgery again were comparable to those with NHL. These findings highlight an association between health literacy and post-operative recovery that likely reflects the influence of broader educational and socioeconomic determinants of health. These findings should be interpreted as associative and hypothesis-generating.

There is growing interest in quantifying the success of orthopedic operations via patient outcomes and satisfaction.^3^ Recent literature has highlighted health literacy as an important factor in post-operative outcomes.^1^^,^^19^^,^^21^^,^^30^ Health literacy is the degree to which patients can obtain, process, understand, and communicate about health-related information needed to make informed health decisions.^2^ Many health-related materials distributed to patients are beyond the reading comprehension level of the majority of Americans.^11^ The Literacy in Musculoskeletal Problems (LiMP) survey was created to assess musculoskeletal (MSK) health.^17^^,^^19^ LiMP was written at a grade level of 4.2 and validated against a general health literacy assessment tool (Newest Vital Sign).^17^^,^^19^

Several studies have demonstrated that low MSK health literacy is associated with inferior health outcomes and reduced satisfaction with care.^4^^,^^5^^,^^12^^,^^15^ Low MSK health literacy has been shown to significantly impact clinical outcomes, patient satisfaction, rehabilitation rates, and opioid consumption in total knee arthroplasty.^5^^,^^10^^,^^19^^,^^32^ There is a paucity of data analyzing health literacy and outcomes in other orthopedic patients, specifically total shoulder arthroplasty (TSA) patients.^9^

The aim of this study was to investigate how MSK health literacy, as measured by LiMP, impacts patient outcomes and satisfaction following elective TSA. We hypothesized that low MSK health literacy score would be associated with inferior clinical outcomes and lower patient satisfaction rates.

## Materials and methods

This study was approved by the local institutional review board prior to data collection.

### Study population

We retrospectively identified all patients at a single institution who underwent primary total shoulder arthroplasty (Current Procedural Terminology code 23472) between 2014 and 2022, including both anatomic total shoulder arthroplasty (aTSA) and reverse total shoulder arthroplasty (rTSA). Patients were included if the indication for surgery was degenerative glenohumeral osteoarthritis, including cases with associated rotator cuff pathology treated with rTSA at the discretion of the treating surgeon. Patients undergoing TSA for nondegenerative indications, including acute fracture, revision arthroplasty, infection, or tumor, were excluded. A minimum post-operative follow-up of 6 months was required, as prior studies in TSA show that most clinically meaningful improvements occur within this period and correlate with longer-term outcomes.^16^^,^^20^^,^^22^^,^^28^ Patients who underwent revision TSA or TSA for proximal humerus fractures were excluded. All patients were treated by one of 2 sport fellowship–trained surgeons. Discharge education protocols did not change over the study period. All patients received standardized written and verbal instructions both at a pre-operative clinic visit and again prior to hospital discharge.

### Survey methods

**Figure 1:**
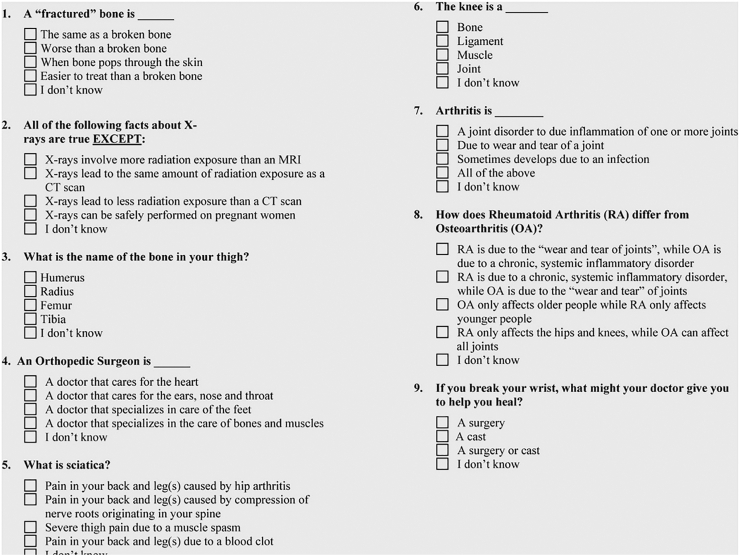
Survey questions included on the LiMP survey, a validated musculoskeletal health literacy scale. *LiMP*, Literacy in Musculoskeletal Problems; *MRI*, magnetic resonance imaging; *CT*, computed tomography.

Patients were sent a one-time survey that included basic demographics, validated MSK health literacy scale (LiMP, Fig. 1), the American Shoulder and Elbow Surgeon (ASES) shoulder score, and a TSA satisfaction questionnaire. The questionnaire asked, using a 5-point Likert scale, how satisfied they were with the results of their surgery and if they would choose to undergo the same operation again. All survey questions included clear written instructions directing participants to select a single response for each item, and all measures were administered using fixed-response or multiple-choice formats. Patients were categorized as having low health literacy (LHL) if they answered fewer than 6 out of 9 questions correctly on the LiMP survey and as having normal health literacy (NHL) if they answered 6 or more questions correctly. Previous research has validated this cutoff as a sensitive and specific indicator for identifying patients with LHL or NHL.^24^^,^^25^

### Demographics and outcomes

The electronic medical record was retrospectively reviewed for each participant to determine, at the time of the survey, age, race, body mass index (BMI), tobacco use status, and type of arthroplasty—reverse (rTSA) or anatomic TSA (aTSA). The primary outcomes of interest were ASES shoulder score and patient satisfaction. Secondary outcomes include post-operative complications (within entire follow-up period), length of stay, discharge disposition, skilled nursing facility length of stay, Charlson-Deyo Comorbidity Index (CCI), American Society of Anesthesiologists physical status classification, and 3-month morphine milligram equivalents (MME). MME was determined from the number of opioid (ie, oxycodone, hydrocodone, tramadol) tablets and prescriptions provided within three months post-operatively.

### Statistical analysis

LHL and NHL groups were compared with Student *t*-tests for continuous variables and Fisher exact tests for categorical variables. Multivariate logistic and linear regression models were used to assess the relationship of MSK health literacy and ASES. The covariates for these models were selected from the bivariate comparisons and included variables that were significant (*P* < .05) or nearly significant (*P* < .05 + 0.025) between the groups. Odds ratios (ORs) with 95% confidence intervals (CIs) were reported. A subgroup analysis comparing anatomic and reverse TSA was performed. Data analysis was conducted with Prism GraphPad (San Diego, CA).

## Results

### Basic demographics

A total of 734 eligible patients were contacted, of whom 310 returned surveys (42% response rate). After excluding patients not meeting inclusion criteria or with incomplete surveys, 230 patients were included in the final analysis. Surveys were considered incomplete if responses to any of the 9 health literacy items were missing or if the ASES or TSA satisfaction questionnaire was left blank. The average LiMP score for the cohort was 6.6 ± 1.5. Median LiMP was 7 with an interquartile range from 6 to 8. One hundred seventy-seven individuals (77.0%) were classified as NHL, and 53 individuals (23.0%) were classified as LHL.

Study follow-up, defined as the time between index surgery and survey completion, ranged from 6 to 110 months post-operatively, reflecting the cross-sectional nature of survey administration in this cohort. The mean follow-up duration was 87.9 ± 64.1 months for the LHL group and 101.8 ± 72.7 months for the NHL group. Of the 230 participants, 215 (93.5%) identified as Caucasian, 10 (4.4%) as African American, 2 (0.87%) as Hispanic, and 3 (1.3%) as other (Table I). There were no significant differences between the NHL and LHL groups in regard to race, age, sex, body mass index, smoking status, CCI, or American Society of Anesthesiologists score (Table I).

**Table I:** Cohort demographics.

Variable	Low health literacy (n = 53)	Normal health literacy (n = 177)	Overall cohort (n = 230)	*P* value
Age (yr, mean ± SD)	71.8 ± 10.9	74.0 ± 8.4	73.5 ± 9.1	.1116
Gender				
Male	25 (47.2%)	70 (39.5%)	95 (41.0%)	.3432
Female	28 (52.8%)	107 (60.5%)	135 (59.0%)	
Race				
White	44 (83.0%)	171 (96.6%)	215 (93.5%)	.4042
Black	6 (11.3%)	4 (2.3%)	10 (4.4%)	
Hispanic	2 (3.8%)	0 (0%)	2 (0.87%)	
Other	1 (1.9%)	2 (1.1%)	3 (1.3%)	
Procedure type				
Anatomic TSA	20 (37.7%)	61 (34.5%)	81 (35.2%)	.7433
Reverse TSA	33 (62.3%)	116 (65.5%)	149 (64.8%)	
BMI (mean ± SD)	30.9 ± 7.1	30.1 ± 6.8	30.3 ± 6.8	.5001
Smoking status				
Never	30 (56.6%)	90 (50.8%)	120 (52.2%)	.5315
Current or former	23 (43.4%)	87 (49.2%)	110 (47.8%)	
Opioid use				
Total MME (mean ± SD)	408.1 ± 341.8	663.0 ± 1,127	604.3 ± 1,007	.1061
Number of opioid prescriptions	1.34 ± 0.55	1.42 ± 0.84	1.40 ± 0.78	.5216
CCI (mean ± SD)	3.55 ± 1.67	3.39 ± 1.52	3.43 ± 1.56	.5203
ASA class (mean ± SD)	2.60 ± 0.57	2.55 ± 0.63	2.59 ± 0.59	.6449

*SD*, standard deviation; *BMI*, body mass index; *MME*, morphine milligram equivalents; *CCI*, Charlson-Deyo Comorbidity Index; *ASA*, American Society of Anesthesiologists; *TSA*, total shoulder arthroplasty.

Eighty-one patients (35.2%) had an anatomic arthroplasty and 149 (64.8%) had a reverse arthroplasty. In the LHL group, there were 20 (37.7%) anatomic and 33 (62.3%) reverse. The NHL group consisted of 61 (34.5%) anatomic and 116 (65.5%) reverse (Table I).

The mean MME for the cohort was 604.3 ± 1,007 (0-8,100). The mean MME for those with LHL was 408.1 ± 341.8 and for those with NHL was 663.0 ± 1,127 (*P* = .106).

### Primary outcome measures

**Figure 2:**
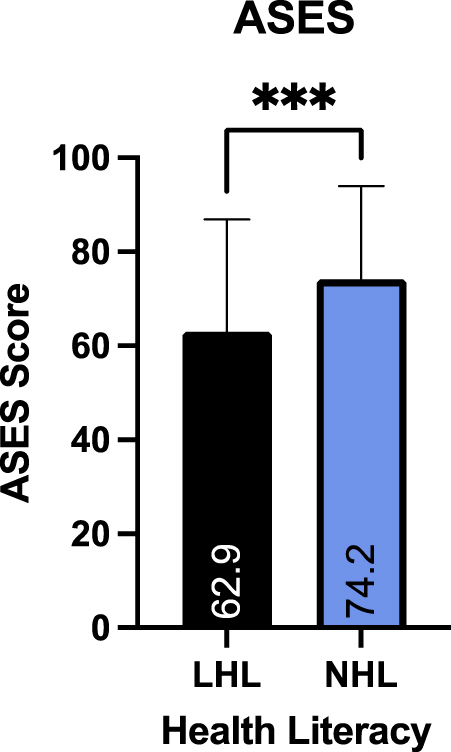
Mean ASES according to health literacy. Those in the normal health literacy group had a significantly higher (74.2 ± 19.8) ASES score compared to those in the low health literacy group (62.9 ± 23.9; *P* = .0007). *ASES*, American Shoulder and Elbow Surgeons; *LHL*, low health literacy; *NHL*, normal health literacy.

**Figure 3:**
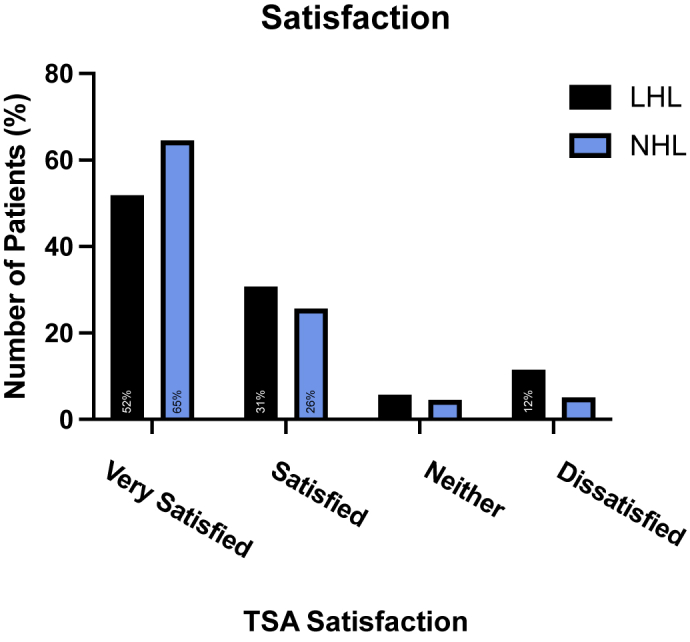
Number of patients satisfied with their TSA by health literacy group. Satisfaction rates were not significantly different between those in the low health literacy and normal health literacy groups (*P* = .106). *TSA*, total shoulder arthroplasty; *LHL*, low health literacy; *NHL*, normal health literacy.

Average ASES score for the cohort was 71.6 ± 21.3. The average ASES score for the NHL group was significantly higher than the LHL group (74.2 ± 19.8 vs. 62.9 ± 23.9*, P* = .0007, Fig. 2). Regarding patient satisfaction, 90.3% of those in the NHL group reported being satisfied or very satisfied compared to 82.7% in the LHL group; however, this was not statistically significant (*P* = .2101, Fig. 3). No statistically significant difference was found in the willingness to undergo the same operation again between the NHL and LHL groups (89.8% vs. 84.9%, *P* = .334).

Eleven of the 230 participants (4.8%) required a rehabilitation stay after discharge from the hospital. Of the patients in the LHL group, 5 of 53 (9.4%) were discharged to a rehab facility compared with 6 of 177 (3.4%) in the NHL group (*P* = .4948). There were 14 patients who required revision surgery—2 (3.8%) in the LHL group and 12 (6.8%) in the NHL group. Patients required revision surgery for periprosthetic fracture (29%), instability (21%), infection (21%), loose components (14%), continued pain (7%), or traumatic base plate dissociation (7%). Rate of revision surgery was not statistically significant between the groups (*P* = .3595). There was no significant difference in rate of complications between aTSA and rTSA (*P* = .4243).

The multivariate linear regression analysis identified MSK health literacy (β, 10.25; 95% CI, 3.861-16.64, *P* = .0018) and discharge to rehab (β, 10.25; 95% CI, 3.861-16.64, *P* = .0018) as being independently predictive of ASES score (Table II). The other covariates included in the model, including gender, procedure type smoking status, and CCI, were not independently predictive of ASES score. There was no significant difference in ASES between aTSA and rTSA (*P* = .3522). The multivariate logistic regression analysis found that higher ASES score (OR, 1.025; 95% CI, 1.010-1.040, *P* = .0009) was independently associated with higher health literacy. The other covariates were not independently associated with higher HL (Table III).

**Table II:** Multivariate linear regression identifying predictors of ASES score in patients undergoing total shoulder arthroplasty.

Variable	Beta coefficient (95% CI)	*P* value
MSK health literacy	10.25 (3.861-16.64)	.0018
Gender	4.270 (−1.233 to 9.773)	.1277
Procedure type	−1.414 (−7.160 to 4.333)	.6283
CCI	−0.328 (−2.059 to 1.403)	.7091
Smoking status	3.046 (−2.419 to 8.511)	.2733
Rehab	−19.25 (−31.97 to −6.528)	.0032

*ASES*, American Shoulder and Elbow Surgeons; *MSK*, musculoskeletal; *CCI*, Charlson-Deyo Comorbidity Index; *CI*, confidence interval.

**Table III:** Multivariate logistic regression identifying predictors of normal health literacy.

Variable	Odds ratio (95% CI)	*P* value
ASES	**1.025 (1.010-1.040)**	**.0009**
Gender	0.647 (0.336-1.241)	.1896
Procedure type	1.174 (0.598-2.276)	.6363
Smoking status	1.206 (0.628-2.334)	.5754
CCI	0.998 (0.817-1.226)	.9879
Rehab	0.539 (0.143-2.123)	.3601

*ASES*, American Shoulder and Elbow Surgeons Score; *CCI*, Charlson-Deyo Comorbidity Index; *CI*, confidence interval.

Bolded values indicate statistical significance (P < 0.05).

### Subgroup analysis

An independent subgroup analysis comparing outcomes based on health literacy in the aTSA group (n = 81) and rTSA group (n = 149) was completed. In the anatomic TSA group, there were 61 patients (75.3%) with NHL and 20 patients (24.7%) with LHL. In the reverse TSA group, there were 116 patients (77.9%) with NHL and 33 patients (22.1%) with LHL. There were no significant differences between NHL and LHL within the aTSA subgroup when comparing ASES (75.8 ± 17.7 vs. 67.7 ± 25.7, *P* = .1408), satisfaction (94.7% vs. 88.2%, *P* = .2098), and willingness to undergo surgery again (86.0% vs. 82.4%, *P* = .7073). In the rTSA subgroup comparing NHL and LHL, there was a significantly higher ASES score (73.6 ± 20.7 vs. 61.2 ± 24.5, *P* = .0076), but no significant differences in satisfaction (90.2% vs. 80.0%, *P* = .1493) and willingness to undergo surgery again (93.0% vs. 83.3%, *P* = .1483).

## Discussion

This study found that 23% of elective TSA patients had LHL, a rate consistent with previously published literature.^21^^,^^27^ In addition, patients in the LHL group demonstrated significantly lower ASES scores at the time of survey compared to those with NHL, suggesting a potential association between health literacy and post-operative function. Multivariate regression analysis demonstrated an association between health literacy and post-operative ASES score after adjustment for available clinical covariates, including comorbidity burden and procedure type. However, important social determinants such as education level and socioeconomic status were not available for analysis and may confound this relationship. While the regression model showed statistical significance, the effect size was modest with an OR of only 1.025. These findings support the idea that while MSK health literacy may not independently drive large clinically significant differences in outcomes, it remains an important and potentially modifiable factor associated with post-operative recovery and patient-reported function after TSA. Given the timing of literacy assessment in this study, these relationships should be interpreted as associative rather than causal.

Although the association between health literacy and ASES score reached statistical significance, the observed effect size was modest. The absolute difference in mean ASES scores between the normal and LHL groups was approximately 11 points. Prior studies have reported Minimal Clinically Important Difference thresholds for the ASES score following shoulder arthroplasty ranging from approximately 6 to 12 points.^20^^,^^29^^,^^31^ As such, the observed difference approaches commonly cited Minimal Clinically Important Difference values but may not uniformly represent a clinically meaningful change for all individual patients. These findings should therefore be interpreted as reflecting a population-level association rather than a definitive predictor of clinically meaningful improvement at the individual level. Despite the modest magnitude of effect, even small shifts in patient-reported outcome scores at the population level may have meaningful implications for health system performance, patient satisfaction, and resource utilization, particularly when applied to high-volume procedures such as TSA. In this context, health literacy may represent a modifiable factor that contributes incrementally to post-operative recovery within a broader constellation of clinical and social determinants.

The subgroup analysis revealed that low MSK health literacy was associated with significantly worse ASES scores in patients undergoing rTSA, whereas no statistically significant association was observed in the aTSA subgroup. Although this finding may initially appear counterintuitive, given the more restrictive rehabilitation protocols traditionally associated with aTSA, it suggests that rTSA may, in certain respects, place greater cognitive and behavioral demands on patients during postoperative recovery. Reverse arthroplasty relies heavily on neuromuscular adaptation and motor reprogramming, as patients must learn to generate shoulder elevation using altered biomechanics and a fixed center of rotation. Patients with LHL may have greater difficulty understanding these biomechanical changes, internalizing therapy goals, or effectively translating rehabilitation instructions into functional movement patterns.

In contrast, recovery following aTSA more closely restores native shoulder anatomy and kinematics, which may allow patients to rely on more intuitive movement patterns despite stricter early post-operative restrictions related to subscapularis or lesser tuberosity healing. In addition, the more prolonged protection phase after aTSA may result in closer supervision by physical therapists and caregivers, potentially mitigating the impact of LHL. Conversely, the relatively rapid functional use permitted after rTSA may place greater responsibility on the patient to self-regulate activity, adhere to nuanced rehabilitation guidance, and adapt psychologically to new biomechanics. These factors may disproportionately challenge patients with LHL and contribute to inferior functional outcomes in this subgroup.

Importantly, these interpretations are speculative and should be viewed as hypothesis-generating. Future studies incorporating detailed rehabilitation adherence data, cognitive or psychosocial measures, and prospective assessment of health literacy may help clarify the mechanisms underlying these subgroup-specific findings.

Health literacy is closely intertwined with broader educational and socioeconomic factors that influence patients' ability to access, process, and act upon health information. As such, the associations observed in this study may reflect the cumulative effects of these upstream determinants rather than health literacy in isolation. In this context, health literacy may function as a pragmatic and clinically accessible marker of vulnerability rather than a singular causal factor driving post-operative outcomes.

Health literacy research within orthopedic surgery has expanded in recent years. Kadakia et al^12^ showed that orthopedic trauma patients often have limited comprehension of injuries and post-operative care instructions. Their analysis revealed that patients with lower educational levels were more likely to have LHL.^12^ Numerous cross-sectional studies have determined the prevalence and risk factors associated with LHL in hand, foot and ankle, and orthopedic trauma patients.^12^^,^^17^^,^^23^^,^^24^^,^^26^ Narayanan et al^19^ further demonstrated the importance of LHL in total knee arthroplasty patients, as it was associated with both inferior clinical outcomes and lower post-operative patient satisfaction rates. To date, only 2 studies in the literature have investigated health literacy in TSA patients.^21^^,^^27^ One study of 90 TSA patients found that just over a third of the study cohort had LHL.^27^ A larger retrospective study of 230 patients demonstrated that 25.2% of TSA patients had LHL. Interestingly, they found that the LHL patients had lower ASES scores, higher visual analogue scale (VAS) pain scores, and extended hospital stays.^21^ Their results lacked external validity because the study was conducted at an orthopedic specialty hospital. Over 70% of patients included had perfect scores on health literacy assessments, thus limiting the generalizability of the results to institutions that treat a full gambit of medical subspecialites.^21^ In our study, aside from the association noted between ASES score and health literacy, there was a trend indicating that patients with LHL experienced lower satisfaction after TSA and higher rates of discharge to rehabilitation facilities. One possible explanation is that patients with LHL may have had a more limited understanding of the surgical process, expected recovery trajectory, or the demands of post-operative rehabilitation. Inadequate comprehension of pre-operative counseling or discharge planning may have led to unmet expectations or increased reliance on extended post-operative support, contributing to both decreased satisfaction and a greater likelihood of discharge to rehabilitation facilities. No significant differences were observed between groups in patient satisfaction or willingness to undergo surgery again. This may, in part, reflect the simplicity of the questionnaire items, which are designed as global assessments and may not capture more subtle differences in patient experience.^7^^,^^13^ Given the high baseline satisfaction typically reported after TSA, ceiling effects could also have limited our ability to detect between-group differences.

The wide range of follow-up duration in this study introduces additional complexity in interpreting patient-reported outcomes. Over long-term follow-up, ASES scores and satisfaction may be influenced by aging, progression of medical comorbidities, contralateral shoulder disease, or unrelated health decline, independent of surgical outcome. As such, outcomes assessed at 6 months are not directly comparable to those assessed many years after surgery, and this heterogeneity likely contributes noise to the observed associations. Although, prior studies in TSA show that most clinically meaningful improvements occur within 6 months and correlate with longer-term outcomes.^16^^,^^20^^,^^22^^,^^28^

Furthermore, patients in the NHL group had a longer mean time between index surgery and survey completion compared to those in the LHL group (101.8 vs. 87.9 months). This difference may reflect increased engagement with the health care system among patients with higher health literacy, potentially contributing to improved long-term outcomes and follow-up survey responsiveness. However, it may also suggest that patients with NHL were more proactive in post-operative care or had fewer barriers to accessing follow-up leading to this discrepancy.

Given the associations observed in this study, there is considerable potential for improvement in the identification of patients with LHL who may benefit from targeted pre-operative interventions. Specifically, by administering the LiMP survey or other validated health literacy assessments during pre-operative appointments, surgeons could proactively identify patients with LHL and target them with interventions that seek to increase their health literacy. This would potentially allow for personalized educational strategies aimed at enhancing patient communication and health comprehension prior to surgery. At our institution, the current standard of care includes providing written pre-operative materials and designating a full clinic visit to review patient expectations and the perioperative care pathway. Prior to discharge, patients receive comprehensive written instructions that are reinforced verbally by nursing and therapy staff. While this structured team approach is intended to standardize the level of understanding among patients, our findings indicate that approximately one quarter of patients undergoing TSA at our institution continued to score in the LHL range, which is concerning. This may be due, in part, to educational materials and delivery methods that are not sufficiently adapted to patients with lower baseline literacy or diverse learning preferences. Written materials, even when reinforced verbally, may still exceed the reading comprehension or processing ability of certain patients, particularly older individuals or those with limited formal education. We believe the educational approach taken at our institution reflects those standard practices employed at many tertiary care centers across the country, yet it still seems to be insufficient in providing adequate patient education. These findings highlight the need to reassess the effectiveness of current educational strategies and consider incorporating supplemental or alternative interventions, such as visual aids, interactive digital health tools, and online patient engagement platforms, including videos and potentially an arthroplasty class. Ultimately, this discrepancy directly increases the risk of worse post-operative clinical outcomes and these findings highlight the need to reassess the effectiveness of current educational strategies. Surgeons should consider incorporating supplemental or alternative interventions, such as visual aids, interactive digital health tools, and online patient engagement platforms. In addition, surgeons can simplify communication, use patient educational materials that do not exceed a sixth grade reading level, and implement the teach-back method. These patient-centric practices can better support individuals with LHL and ultimately improve surgical outcomes.

As health care shifts toward more bundled payment reimbursement models, hospitals and medical practices must continue to optimize system efficiency, cost reduction, and improved patient-centered outcomes. LHL has been identified as an important modifiable social determinant of health that impacts multiple aspects of perioperative care. Future studies in larger and more diverse patient populations should prospectively investigate processes and interventions to maximize health literacy as part of pre-operative optimization before TSA surgery to better understand its influence on post-operative outcomes.

### Limitations

This study has several limitations. First, the LiMP survey was administered at different time points post-operatively; therefore, the health literacy survey results may not accurately reflect pre-operative health literacy levels or a consistent post-operative health literacy given the large range we observed. All surveys were administered electronically via email, which introduces the potential for selection bias related to digital literacy. Patients with higher digital literacy are more likely to respond to electronic surveys, and digital literacy likely correlates with health literacy.^8^^,^^18^ As a result, patients with the lowest health literacy may be under-represented in this cohort due to nonresponse rather than exclusion. This bias may lead to an underestimation of both the prevalence and impact of limited health literacy on post-operative outcomes following TSA. Fewer than 20 surveys were returned as undeliverable, and these were not resent through alternative modalities, which may have contributed to nonresponse bias. Moreover, electronic self-administration without opportunities for in-person clarification could have posed challenges for some patients with limited health literacy. Nevertheless, electronic administration mirrors contemporary clinical practice and enhances the generalizability of our findings to real-world outpatient settings, where digital survey tools are increasingly utilized. In addition, all surveys were administered in English and required only fixed-response or multiple-choice answers. Nevertheless, non–English-speaking patients were not represented in this cohort, which may limit generalizability and potentially underestimate the impact of limited health literacy in more linguistically diverse populations. Future studies should incorporate multimodal survey strategies, including paper-based questionnaires, telephone follow-up, or in-person administration, to better capture patients with limited digital access and literacy and to more accurately assess the relationship between health literacy and post-operative outcomes.

Education level and socioeconomic status are well-established determinants of both health literacy and post-operative outcomes and represent important sources of potential residual confounding in this study. Because these variables were not consistently available in our dataset, we were unable to adjust for their effects or assess their relative contribution to the observed associations. As a result, it is not possible to determine whether health literacy itself is the primary driver of differences in patient-reported outcomes or whether it serves as a proxy for broader educational and socioeconomic disadvantage. Future studies incorporating individual-level educational attainment and validated socioeconomic indices, such as the Area Deprivation Index or Social Vulnerability Index, are needed to clarify these relationships.

The absence of pre-operative assessments (LiMP surveys and ASES scores) prevents the evaluation of functional improvement compared to baseline. While our study combined the patient populations of 2 surgeons who have similar pre-operative and post-operative protocols, subtle differences, such as operative technique or implant selection, may have influenced outcomes. Furthermore, our survey was sent to patients through email, which could have led to a selection bias by targeting patients with internet access and higher digital literacy.

A key limitation of this study is that health literacy was assessed at the time of survey completion rather than pre-operatively, with survey administration occurring up to nine years following surgery. As such, the measured health literacy may not accurately reflect patients' literacy at the time of surgery and may instead represent literacy influenced by aging-related cognitive changes, evolving health status, or post-operative experiences and engagement with the health care system. While prior research suggests that health literacy in adults is generally stable over time, gradual declines have been observed with aging and cognitive change.^6^^,^^14^ Therefore, literacy status at the time of surgery may not have been identical to literacy status at the time of survey, which complicates interpretation of the association between health literacy and outcomes. Consequently, the observed associations between health literacy and outcomes should be interpreted cautiously and viewed as exploratory. Future prospective studies incorporating pre-operative baseline health literacy assessment are needed to better define the temporal and causal relationship between health literacy and post-operative outcomes following TSA.

Another important limitation of this study is the heterogeneous follow-up duration, which ranged from 6 to 110 months. Patient-reported outcomes collected over such a broad time span may be influenced by factors unrelated to the index procedure, including aging, progression of comorbid conditions, contralateral shoulder pathology, and general health decline. This variability limits direct comparability across patients and likely introduces noise that may confound observed associations. Importantly, such heterogeneity would be expected to bias results toward the null, suggesting that the observed association between health literacy and ASES score may be conservative. Future prospective studies with standardized follow-up intervals or predefined long-term assessment windows are needed to better characterize the durability and clinical significance of these findings.

While we required a minimum of 6 months based on prior evidence that most recovery occurs within this time frame, longer and more uniform follow-up intervals would provide greater consistency and allow for stronger assessment of the durability of outcomes.^16^^,^^20^^,^^22^^,^^28^ Lastly, our study was conducted at a large tertiary academic medical center in the southeastern United States, which may limit the generalizability of our results to practices in other locations or settings. Especially given that the study cohort was composed largely of Caucasian patients, which does not fully represent the demographics of our institution. This discrepancy may reflect underlying disparities in which patients undergo TSA and which patients respond to electronic surveys. As such, the racial composition of our sample limits the generalizability of these findings and may introduce bias related to access to surgery, health literacy, and post-operative outcomes.

## Conclusion

This study identified approximately one-quarter of patients undergoing TSA at our institution had LHL. Patients with LHL were found to have significantly lower patient-reported outcome scores, although rates of patient satisfaction and willingness to undergo surgery again were comparable to those with NHL. These findings highlight an association between health literacy and post-operative recovery that likely reflects the influence of broader educational and socioeconomic determinants of health. These findings should be interpreted as associative and hypothesis-generating, underscoring the need for prospective studies that assess health literacy in the pre-operative period.

## Disclaimers:

Funding: No funding was received for this study.

Conflicts of interest: The authors, their immediate families, and any research foundations with which they are affiliated have not received any financial payments or other benefits from any commercial entity related to the subject of this article.
